# Identification of hepatocellular carcinoma prognostic markers based on 10-immune gene signature

**DOI:** 10.1042/BSR20200894

**Published:** 2020-08-28

**Authors:** Kaifei Zhao, Lin Xu, Feng Li, Jin Ao, Guojun Jiang, Rongshu Shi, Fang Chen, Qing Luo

**Affiliations:** 1 Department of Intervention, The Affiliated Hospital of Zunyi Medical University, Zunyi, Guizhou Province 563003, China; 2 Department of Rehabilitation Medicine, The Affiliated Hospital of Zunyi Medical University, Zunyi, Guizhou Province 563003, China; 3 Departments of Cancer Research Laboratory, The Affiliated Hospital of Zunyi Medical University, Zunyi, Guizhou Province 563003, China

**Keywords:** 10-gene signature, bioinformatics, Hepatocellular Carcinoma, immune related genes, TCGA

## Abstract

**Background:** Due to the heterogeneity of hepatocellular carcinoma (HCC), hepatocelluarin-associated differentially expressed genes were analyzed by bioinformatics methods to screen the molecular markers for HCC prognosis and potential molecular targets for immunotherapy.

**Methods:** RNA-seq data and clinical follow-up data of HCC were downloaded from The Cancer Genome Atlas (TCGA) database. Multivariate Cox analysis and Lasso regression were used to identify robust immunity-related genes. Finally, a risk prognosis model of immune gene pairs was established and verified by clinical features, test set and Gene Expression Omnibus (GEO) external validation set.

**Results:** A total of 536 immune-related gene (IRGs) were significantly associated with the prognosis of patients with HCC. Ten robust IRGs were finally obtained and a prognostic risk prediction model was constructed by feature selection of Lasso. The risk score of each sample is calculated based on the risk model and is divided into high risk group (Risk-H) and low risk group (Risk-L). Risk models enable risk stratification of samples in training sets, test sets, external validation sets, staging and subtypes. The area under the curve (AUC) in the training set and the test set were all >0.67, and there were significant overall suvival (OS) differences between the Risk-H and Risk-L samples. Compared with the published four models, the traditional clinical features of Grade, Stage and Gender, the model performed better on the risk prediction of HCC prognosis.

**Conclusion:** The present study constructed 10-gene signature as a novel prognostic marker for predicting survival in patients with HCC.

## Introduction

Hepatocellular Carcinoma (HCC) is the sixth most common morbidity in the world, and the third most common malignant tumor [1], is also the leading cause of death in patients with cirrhosis [2]. There are more than 500,000 new cases worldwide each year, of which China accounts for 55% [1]. With the continuous development and improvement of imaging, biochemical, pathological and other technical means in recent years, early diagnosis and treatment of HCC have made great progress, and the survival rate has also been significantly improved. But for the entire population of HCC patients, the overall 5-year survival rate is still less than 5% [3], the most important reason is the extremely high metastatic recurrence rate of HCC. It is found that even in patients with radical resection, the 5-year metastasis recurrence rate is as high as 60–70%, and local treatment is higher [4]. Therefore, in-depth study of the mechanism of HCC metastasis and recurrence, to find effective indicators to predict tumor recurrence, active prevention and timely treatment of recurrence is the key to improve the overall efficacy of HCC patients.

The immune imbalance in the tumor microenvironment is one of the important features of the tumor. The adaptive immune response mediated by immune cells plays an extremely important role in the development of tumors [5,6]. The liver as a special immune organ has a unique immune system and participates in the local and overall level of immune regulation. Although the relationship between immunocompetent cells and HCC in the microenvironment of HCC has not been systematically and comprehensively studied, in recent years, the number, distribution and functional status of various types of immune cells in the local microenvironment of HCC have emerged. For example, reporter shows that CDs+ tumour-infiltrating lymphocytes (TILs) can induce apoptosis of HCC cells, thereby controlling the progression of tumors [7]. Cai et al. [8] reported that the more mature dendritic cells (DCs) in the liver microenvironment, the lower the risk of postoperative recurrence and metastasis. If the CDs+CTL or memory T cells with more local infiltration are combined, the risk of recurrence and metastasis is further reduced. Lin et al. [9] reported that activated macrophages enhance the invasion of HCC through adhesion and destabilization. Therefore, it is of great significance to explore the characteristics of immune cells in the HCC microenvironment that affect tumor malignant phenotype, invasion and metastasis ability for the diagnosis, staging and classification of HCC, targeted therapy, prevention of metastasis and recurrence, and improvement of efficacy.

In the present study, immune genes was significantly associated with the prognosis of patients with HCC were determined. Through the feature selection of Lasso, a robust IRGs were finally obtained and a prognostic risk prediction model was constructed. The risk scores of each sample were calculated based on the risk model and divided into high risk group (Risk-H) and low risk group (Risk-L), and the risk model stratified the samples in the training set, test set, external validation set, staging and subtype, and established Kaplan–Meier (KM) curve and receiver operating characteristic (ROC) curve. At the same time, compared with the published four models, the traditional clinical features of Grade, Stage and Gender, the model predicts the risk prediction performance of HCC prognosis.

## Materials and methods

### Data collection

RNA-seq data samples and clinical follow-up information of 371 samples HCC patients were downloaded using the The Cancer Genome Atlas (TCGA) GDC API at August 28, 2019. The set of genes expressed by immune cells was sorted out from the related literatures of immune cell research [10], after removing the genes with repeated names, a total of 3658 genes were found. To be specific, in addition to the collection of immune cell-associated genes from solid tumors, Ajit J. Nirmal et al. identified not only some of the immune cell-associated genes, but also immune cell-associated genes from previous studies.

Gene Expression Omnibus (GEO) data was downloaded from GEO to select the GPL3921 platform in the GSE14520 chip data set with survival time, which contained the Expression data of 445 HCC tissue samples.

ICGC data were downloaded from the HCCDB database, and the HCCDB18 data set with survival time was selected, which contained the expression data of 212 HCC tissues.

### Data preprocessing

The following steps were performed on the RNA-Seq data of 371 samples TCGA-HCC:

1. Samples without clinical follow-up information were removed.
2. Samples without overall suvival (OS) data were removed.
3. The sample without the status were removed.
4. The gene with fragments per kilobase of exon model per million mapped reads (FPKM) <1 in more than half of the samples were removed.
5. Only retain the expression profile of immune cell-related genes.

The following steps for the GEO dataset and the ICGC dataset:

1. The normal tissue sample were removed.
2. Samples without clinical follow-up information were removed.
3. Samples without OS data were removed.
4. The sample without the Status status were removed.
5. For GEO data, the gene with log2 of RMA-calculated Signal intensity <1 in more than half of the samples were removed.
6. For ICGC data, genes with FPKM <1 in more than half of the samples were removed.

Among them, TCGA-HCC has 364 samples, including 1634 genes; GSE14520 has 220 samples as model independent verification set; ICGC has 212 samples, as model independent verification set. The 364 samples of TCGA were divided into two groups, which were similar in age distribution, clinical stage, follow-up time and patient death rate, and the number of samples in the two groups was close to the number of samples. One group is used as the training set (*N*=181), one group is used as the verification set (*N*=183), and the clinical information of the sample is shown in Table 1.

**Table 1:** Sample information

Clinical features	TCGA-LIHC	TCGA-train	TCGA-test	GSE14520	ICGC-LIRI-LP
Event					
Alive	234	120	114	136	176
Dead	130	61	69	84	36
Gender					
M	245	125	120	190	162
F	119	56	63	30	50
T					
T1	179	91	88		33
T2	91	51	40		102
T3	78	34	44		61
T4	13	4	9		16
TX	3	1	2		
N					
N0	248	124	124		
N1	4	2	2		
NX	112	55	57		
M					
M0	263	137	126		
M1	3	2	1		
Mx	98	42	56		
Stage					
I	169	89	80	93	
II	84	48	36	77	
III	83	35	48	48	
IV	4	2	2	0	
X	24	7	17	2	
Grade					
G1	54	21	33		
G2	175	95	80		
G3	118	54	64		
G4	12	8	4		
GX	5	3	2		
Age					
0–50	68	34	34	96	15
50–60	97	46	51	82	24
60–70	116	59	57	31	74
70–100	83	42	41	11	99

### Univariate survival analysis

Univariate Cox proportional risk regression model was developed for each immune-related gene and survival data for training set data. Survival coxph function was used as the R package, and *P*<0.05 was selected as the threshold.

### Construction of prognostic immune gene signature

LASSO is a popular method for regression modeling with a large number of potential prognostic features, because it can perform automatic feature selection in a manner that results in signatures with generally good prognostic performance [11]. The Lasso method has been extended to the Cox model for survival analysis and has been successfully applied for the purpose of building sparse signatures for survival prognosis in many application areas including oncology [12–14]. First, univariate Cox proportional hazards regression analysis was performed on each IRGs using the training set samples. Log rank *P*<0.05 was used as a threshold to identify prognostic IRGs. The R package glmnet [15] was used to screen the robust prognostic features of IRGs, and the multivariate Cox regression analysis was further performed using stepwise regression, and the following risk scoring model was constructed:

$$RiskScore=\sum_{k=1}^{n}Exp_{k}\times eH^{R}k$$

Where *N* is the number of prognostic IRGs, *Exp_k_* is the IRG value of prognostic IRGs, and *e^HR^_k_* is the estimated regression coefficient of IRGs in the multivariate Cox regression analysis.

### Functional enrichment analyzes

Gene Ontology (GO) and Kyoto Encyclopedia of Genes and Genomes (KEGG) pathway enrichment analysis was performed using the R package clusterprofiler [16] for genes, to identify over-represented GO terms in three categories (biological processes, molecular function and cellular component), and KEGG pathway. For this analysis, a FDR  < 0 .05 was considered to denote statistical significance.

Single sample gene set enrichment analysis(ssGSEA) was performed by the R package GSVA [17] using the MSigDB [18] C2 Canonical pathways gene set collection.

### Statistical analysis

The Kaplan–Meier (KM) curve was plotted when the median risk score in each data set was used as a cutoff to compare the risk of survival between the high risk group and the low risk group. Multivariate Cox regression analysis was performed to test whether IRGs markers are independent prognostic factors. Significance was defined as *P*<0.05, both of which were two-sided tests. The ROC analysis uses the R package pROC, and the C-index calculation uses the R package RMS. All of these analyses, if not specified, use default parameters, all in R 3.4.3.

## Results

### Identification of immune-related genes for prognosis differences in patients with HCC

**Figure 1:**
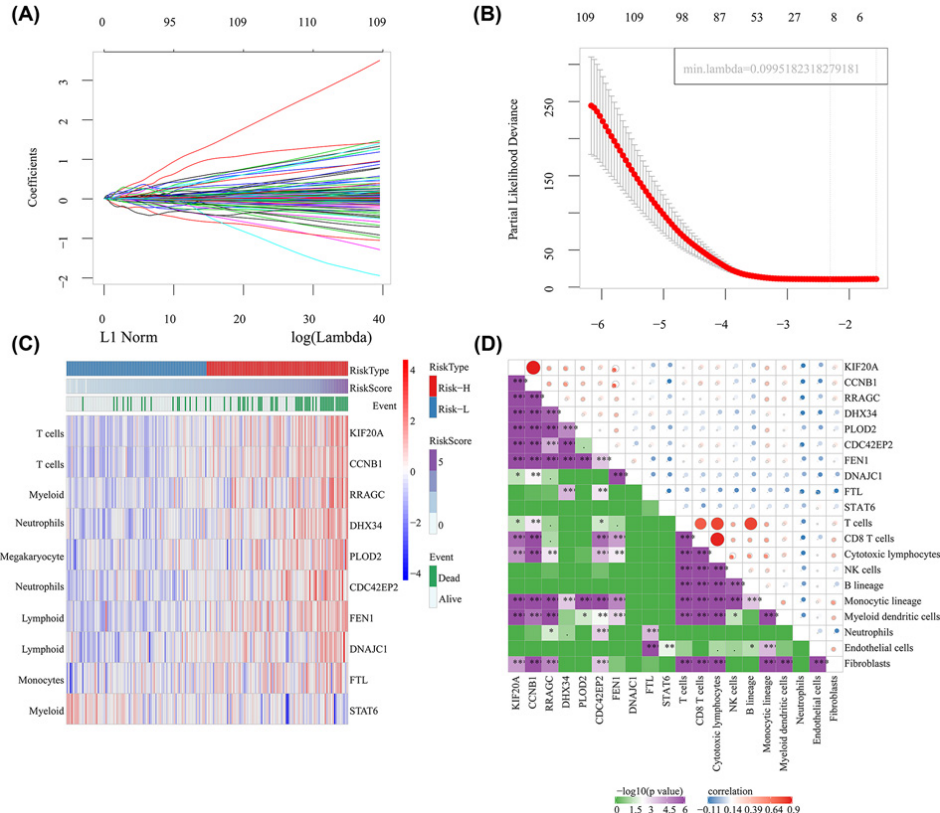
Identification of immune-related genes for prognosis differences in patients with HCC (**A**) The trajectory of each independent variable, the horizontal axis represents the log value of the independent lambda, and the vertical axis represents the coefficient of the independent variable. (**B**) Confidence interval under each lambda. (**C**) Expressions of 10 genes in the training set samples. (**D**)10 genes were highly correlated with the immune cells.

Univariate Cox proportional hazards regression model was used to establish the relationship between the overall survival of patients and the expression of immune-related genes. Finally, there were 536 genetic genes with differences (Supplementary Figure S1). To screen robust immune-related prognostic genes, lasso cox regression was used to perform dimensionality reduction analysis on these 536 genes, resulting in 10 genes (Figure 1A,B). Nine genes (except STAT6) were all more expressed in the high-risk group than in the low-risk group. These 10 genes belong to six types of immune cells: genes KIF20A and CCNB1 are biomarkers of T cells; genes RRAGC and STAT6 are biomarkers of bone marrow cells; genes DHX34 and CDC42EP2 are biomarkers of neutrophils; genes FEN1 and DNAJC1 are biomarkers of lymphocytes; genes PLOD2 are biomarkers of macrophages, and gene FTL is biomarkers of monocytes (Figure 1C). Furthermore, 10 kinds of immune cell scores were evaluated using MCPCounter package, and the relationship between these 10 genes and immune cells was estimated. Similar to previous studies, these 10 genes were highly correlated with the immune cells from their respective sources, among which KIF20A and CCNB1 were significantly correlated with T cells. RRAGC was significantly associated with bone marrow cells. CDC42EP2 had a significant correlation with NEutrophil granulocytes. STAT6 had the best correlation with FTL in ROENTnet cells. DHX34, FEN1 and PLOD2 all had the highest significance in Monocytic lineage (Figure 1D).

### 10-gene signature was established

**Figure 2:**
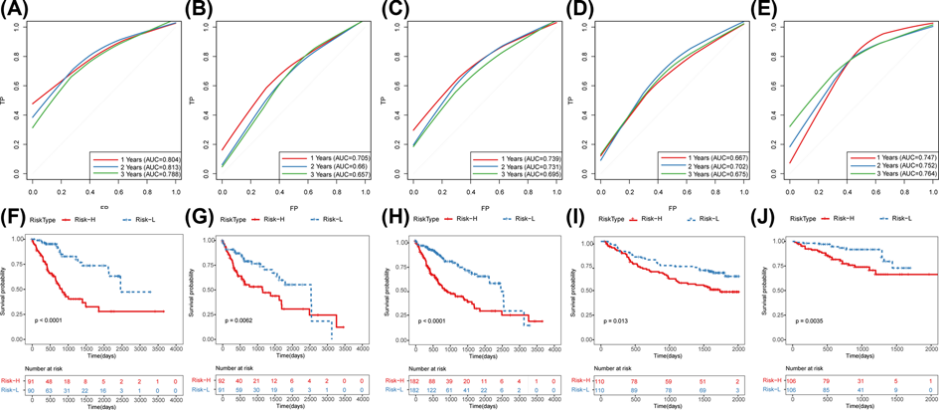
10-gene signature was established (**A**) ROC curve of 10-gene signature model in TCGA training set. (**B**) ROC curve of 10-gene signature model in TCGA validation set. (**C**) ROC curve of 10-gene signature model in TCGA complete set. (**D**) ROC curve of 10-gene signature model in GSE14520 independently validates set. (**E**) ROC curve of 10-gene signature model in ICGC independently validates set. (**F**) KM survival curve of 10-gene signature model in TCGA training set. (**G**) KM survival curve of 10-gene signature model in TCGA validation set. (**H**) KM survival curve of 10-gene signature model in TCGA complete set. (**I**) KM survival curve of 10-gene signature model in GSE14520 independently validates set. (**J**) KM survival curve of 10-gene signature model in ICGC independently validates set.

Risk model of 10 genes was obtained using stepwise regression: RiskScore=KIF20A*0.0324668886039653+CCNB1*0.00128931078300847+RRAGC*0.0603769837171793+DHX34*0.0722652605543841+PLOD2*0.0185191520991944+CDC42EP2*0.0742033718995629+FEN1*0.00107102004808631+DNAJC1*0.00706147977649806+FTL*(3.27178209579064e-06+STAT6*(-0.00483954168677803). Based on the Risk model, the Risk Score of each sample was calculated, and the median Risk Score was taken as the threshold to divide the training set samples into the high-risk group (risk-H) and the low-risk group (risk-L). Considering that the overall distribution of the sample OS is about 2 years (Supplementary Figure S2), the prediction effect of the model ROC in 1, 2 and 3 years is evaluated, with the average AUC∼0.802 (Figure 2A). The KM survival curves of the high-risk group and the low-risk group on the TCGA training data set showed significant differences (Figure 2F).

To verify the stability and reliability of the model, the expression values of the 10 genes in different validation data sets were substituted into the model for verification, the risk score of each sample was calculated, and the risk model ROC and KM curve of 10-gene signature was established.

The verification results of TCGA validation data set showed the average AUC∼0.674 (Figure 2B), and the KM survival curve showed a significant difference (Figure 2G).

The verification results of TCGA complete data set showed the average AUC∼0.722 (Figure 2C), and the KM survival curve showed a significant difference (Figure 2H).

The verification results of independently verify data set GSE14520 showed the average AUC∼0.681 (Figure 2D), and the KM survival curve showed a significant difference (Figure 2I).

The verification results of independently verify data set ICGC-LIRI-JP showed the average AUC∼0.754 (Figure 2E), and the KM survival curve showed a significant difference (Figure 2J).

To evaluate the influence of random sampling on the stability of the model, 1000 random samples of all TCGA samples in different proportions were carried out to evaluate the number of times that the prognosis of the risk-H/risk-L samples in these 1000 random samples was significantly different at each sampling ratio. We found that 995 out of 1000 random samples were significantly different at a sampling ratio of 0.5 (Supplementary Figure S3), which indicated that the risk model of 10 genes established by our random sampling had a relatively low sampling deviation.

### Relationship of RiskScore and pathway

**Figure 3:**
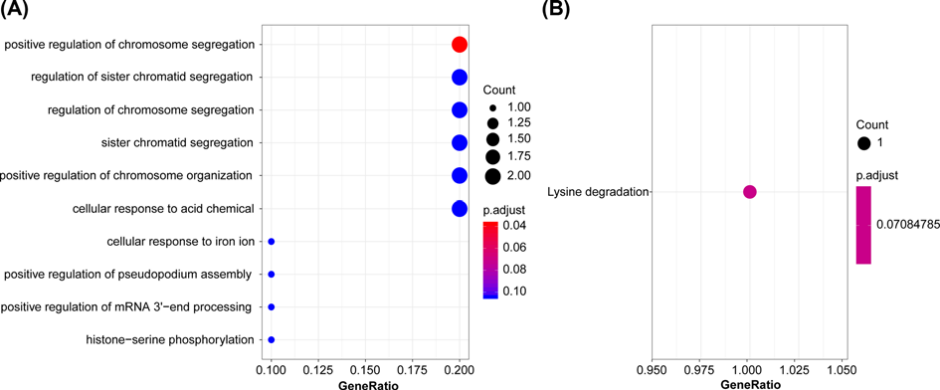
Functional enrichment analysis of 10 genes (**A**) GO enrichment results of 10 genes. (**B**) KEGG enrichment results of 10 genes.

**Figure 4:**
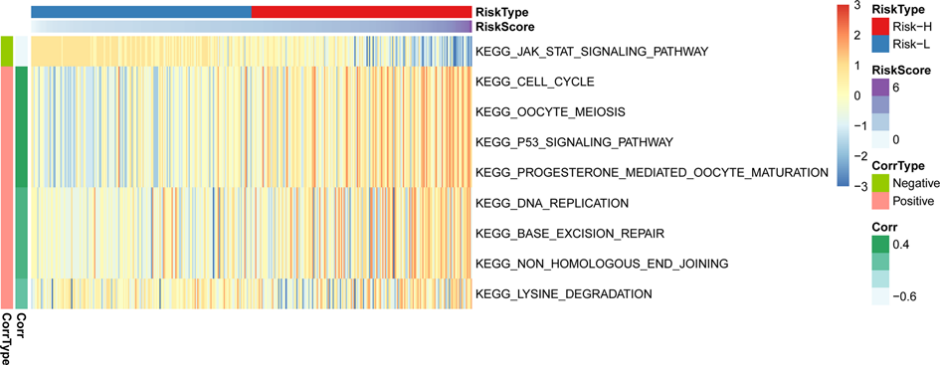
ssGSEA analysis the relationship between RiskScore and KEEG pathway in TCGA training set data

First, GO and KEGG were used for further enrichment analysis of 10 genes, and the results show that it is related to positive regulation of chromosome segregation (Figure 3A), and KEGG pathway enrichment was not significant (Figure 3B). Further, the ssGSEA function analyzed the KEGG function enrichment scores of all TCGA data sets, and the correlation between each Pathway and RiskScore was calculated according to the enrichment scores of each Pathway in each sample, and a total of nine related KEGG pathways were obtained. Clustering analysis was performed on the enrichment scores of nine pathways on all sample sets (Figure 4), and it can be seen that RiskScore is significantly correlated with cell cycle, JAK-STAT and other signaling pathways.

### Analysis of risk models and clinical characteristics

**Figure 5:**
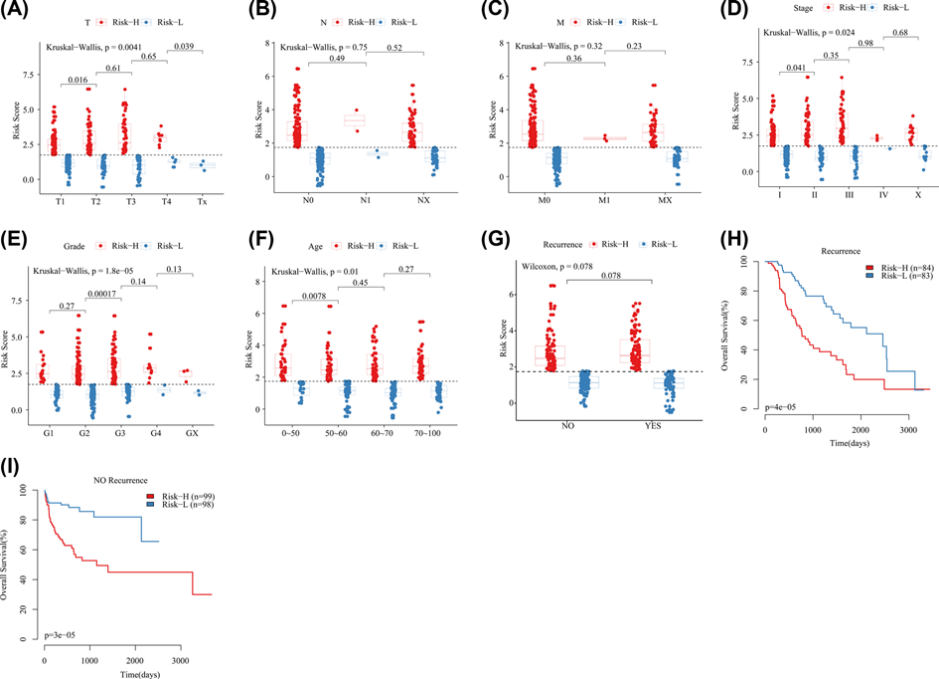
Risk score distribution of clinical features (**A**) Risk score distribution of T stages. (**B**) Risk score distribution of N stages. (**C**) Risk score distribution of M stages. (**D**) Risk score distribution of Stage stages. (**E**) Risk score distribution of Grade stages. (**F**) Risk score distribution of age stages. (**G**) Risk score distribution of Recurrence stages. (**H**) KM survival curve of 10-gene signature model in Recurrence samples. (**I**) KM survival curve of 10-gene signature model in NO Recurrence samples.

Further, the relationship between different clinical characteristics such as T, N, M, Stage, Grade and Age and RiskScore was analyzed (Figure 5), and found that there was no significant difference in the risk scores of samples grouped by clinical features such as N and M, indicating that our risk model was relatively independent of these clinical features. However, there were significant differences in the risk scores of the samples in clinical characteristics groups including T, Stage, Grade and Age (*P*<0.05), indicating that our risk model was correlated with these clinical characteristics. Furthermore, the results of the study on 10-gene signature and HCC recurrence observed that the risk score of patients with liver cancer recurrence was significantly higher than that of patients without recurrence (Figure 5G), in addition, the risk score could significantly divide patients with and without recurrence into high and low risk groups (Figure 5H,I), suggesting that 10-gene signature may be the potential sign of HCC. To prove the broad spectrum of gene signatures, The scores of 10 immune cells from the data sets TCGA, GSE14520 and ICGC-LIRI-LP were evaluated using the MCPCounter package. By comparing the scores of normal and tumor samples, it is found that: (1) in the TCGA data set, the immune score of T cells and lymphocytes cytotoxic is significantly higher than that of normal samples, while the immune score of NK cells, neutrophils and thirdly cytotoxic lymphocytes is significantly lower than that of normal samples, and there is no difference in the scores of the other five immune cells. (2) In ICGC data set, the immune score of tumour samples is significantly higher than that of normal samples, which is consistent with the TCGA data set, while the B dendritic cells, NK cells, neutrophils, CD8 T cells, fibroblasts, and thirdly cytotoxic lymphocytes are significantly lower than that of normal samples. There was no difference in the scores of the other two kinds of immune cells, T cells and monocytic lineage. (3) In the GSE14520 data set, the scores of these 10 immune cells in the normal sample were significantly higher than those in the tumor sample (Supplementary Figure S4). These data indicate that the cells with higher scores of immune cells in normal samples account for more than tumor samples, which indicates that the decline in the immune ability of tumor samples is a potential factor for the development of tumor deterioration.

### RiskScore and clinical features were used to construct the nomogram model

**Figure 6:**
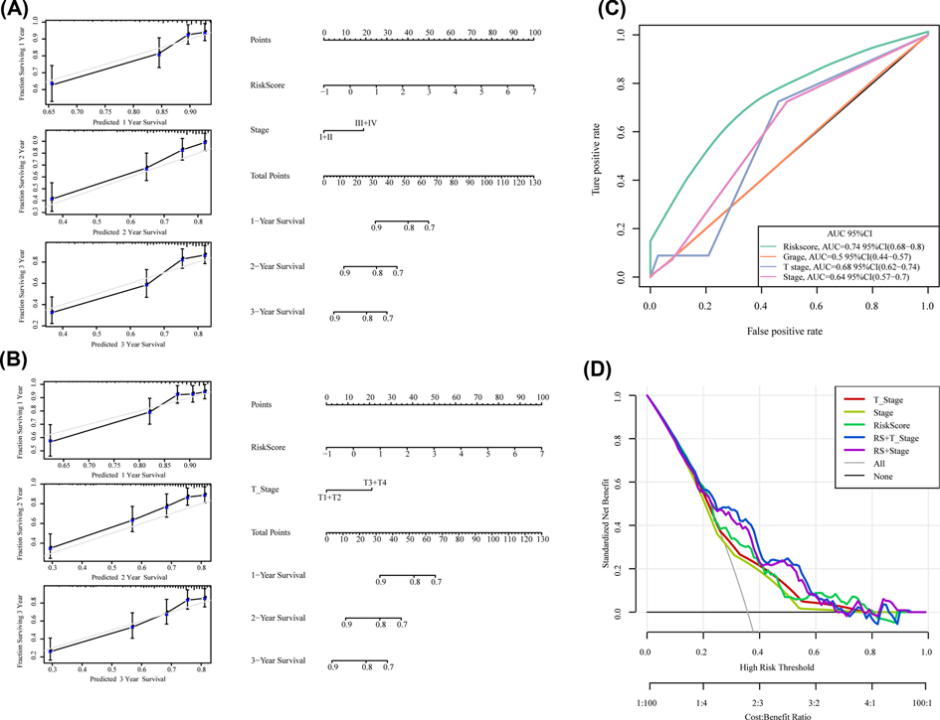
RiskScore and clinical features were used to construct the nomogram model (**A**) The nomogram constructed by Stage+Gender+Grade+RiskScore. (**B**) The nomogram constructed by T+Grade+Gender+RiskScore. (**C**) ROC curver of Stage, T stage, Grade, RiskScore. (**D**) The DCA curve of risk model with clinical features (RiskScore+Stage and RiskScore+T_Stage).

**Figure 7:**
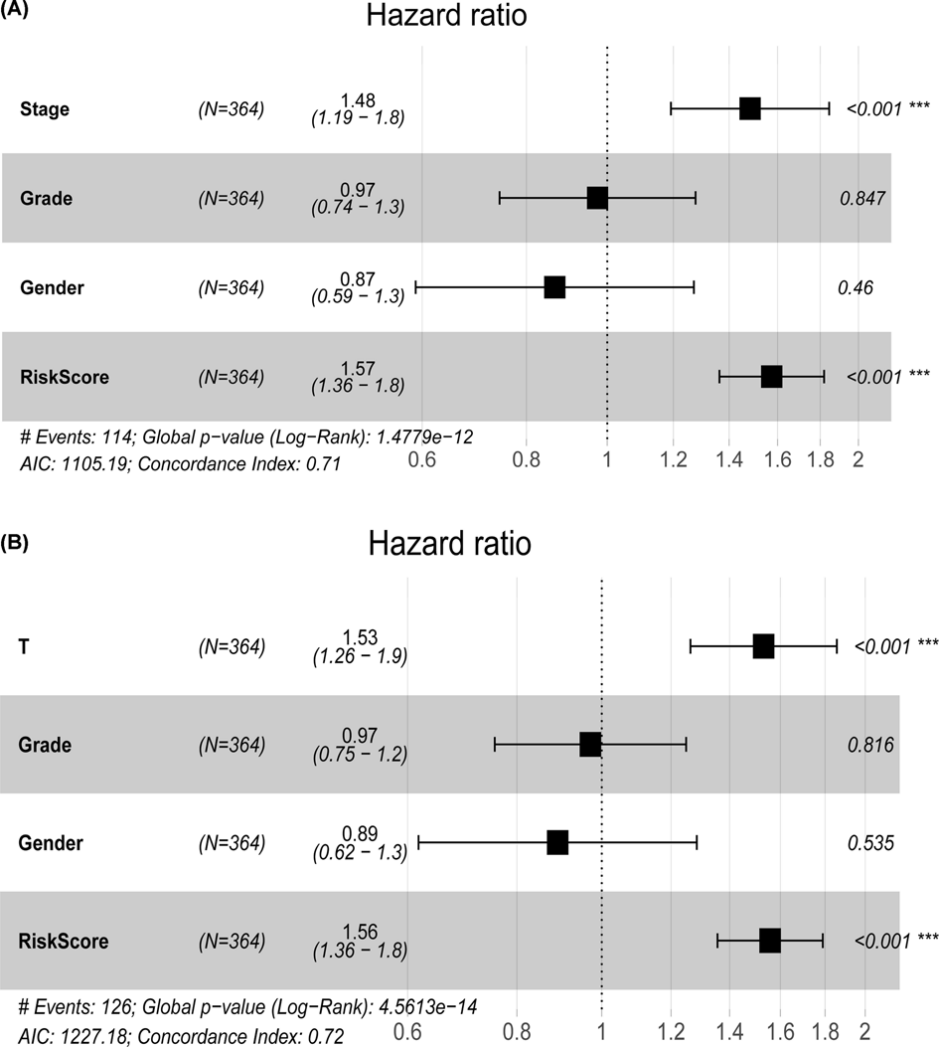
Forest map of RiskScore and clinical features (**A**) The forest map constructed by Stage+Grade+Genderr+RiskScore. (**B**) The forest map constructed by T+Grade+Genderr+RiskScore.

Since there are many missing N and M data in the samples, Grade and Gender are not significant in univariate and multifactor analysis, so they are not included in the analysis. There is inconsistency between T Stage and Stage Stage, so we used all the data sets of TCGA to construct a line graph for the combination of Stage+RiskScore and T+ RiskScore, respectively (Figure 6A,B). According to the model results, RiskScore features have the greatest impact on survival rate prediction, indicating that the risk model based on 10 genes could better predict the prognosis. In order to compare the advantages of clinical features and risk models, ROC curves of clinical features such as Grade, Stage and T Stage were compared with the RishScore model. The results showed that RiskScore had a high AUC value (0.74) (Figure 6C). The DCA curve also shows that RiskScore benefit is higher than the extreme curve. Meanwhile, the integrated models combining the risk model with clinical features (RiskScore+Stage and RiskScore+T_Stage) have a larger curve area (Figure 6D), indicating that the risk model combined with clinical features will have a better effect. Furthermore, clinical features T staging, Stage and RiskScore calculated by risk model were displayed by forest map (Figure 7), displaying the HR values of RiskScore are all around 1.56, and *P*<0.001.

Univariate and multivariate Cox regression analysis (Table 2) was performed on some clinical features and risk models of all TCGA data sets, indicating that our model was not affected by clinical features and had stability.

**Table 2:** Univariate and multivariate Cox analysis data statistics of all TCGA data sets

Variables	Univariable analysis				Multivariable analysis			
	HR	95% CI of HR		*P*	HR	95% CI of HR		*P*
		lower	upper			lower	upper	
**Gender**								
Female								
Male	0.817	0.574	1.165	0.264	0.892	0.608	1.309	0.558
**T Stage**								
T1 + T2								
T3 + T4	2.533	1.78	3.603	2.40E-07	1.739	0.236	12.816	0.587
**Stage**								
I + II								
III + IV	2.441	1.684	3.538	2.50E-06	1.316	0.179	9.673	0.787
**Grade**								
G1 + G2								
G3 + G4	1.116	0.778	1.6	0.551	0.985	0.67	1.45	0.94
**Riskscore**								
Low risk								
High risk	2.63	1.821	3.799	2.60E-07	2.449	1.63	3.677	1.60E-05

### Performance of risk models in different stages and subtypes

**Figure 8:**
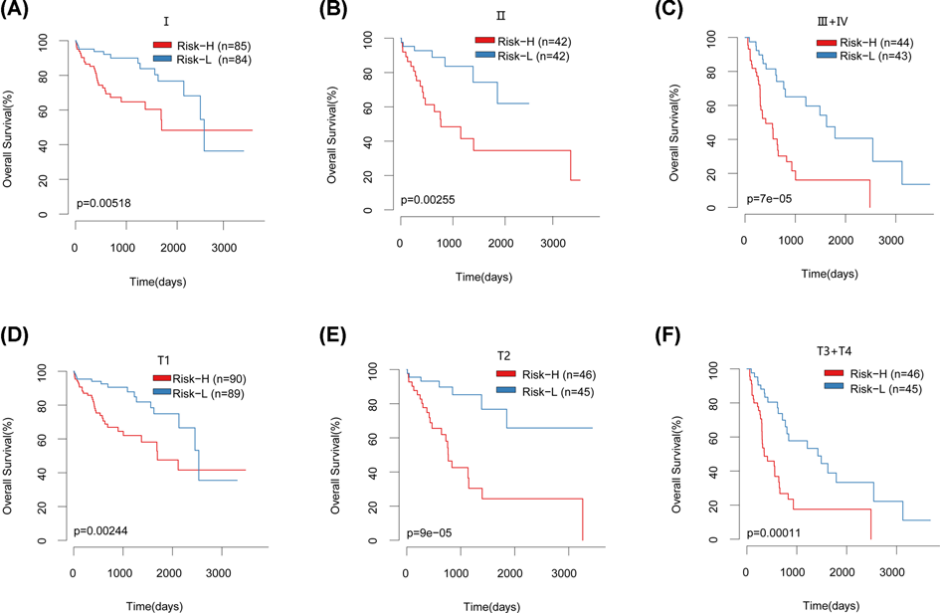
KM suvival curvers of clinical features in TCGA datasets (**A**) KM survival curve of clinical phase I of all datasets of TCGA. (**B**) KM survival curve of clinical phase II of all datasets of TCGA. (**C**) KM survival curve of clinical phase III+IV of all datasets of TCGA. (**D**) KM survival curve of clinical phase T1 of all datasets of TCGA. (**E**) KM survival curve of clinical phase T2 of all datasets of TCGA. (**F**) KM survival curve of clinical phase T3+T4 of all datasets of TCGA.

In order to validate the effect of our model on clinically different staging samples, all data sets of TCGA were classified according to different stages, and our model was used to plot survival curves for samples of different stages. Among the different clinical staging samples, the high-risk and low-risk group of clinical Phase I, Phase II, and III+IV samples had significant differences (Figure 8A–C). High-risk and low-risk groups of clinical T1 staging, T2 staging, and T3+T4 staging samples have significant differences (Figure 8D–F). This shows that our model also has a good effect on different clinical staging samples.

**Figure 9:**
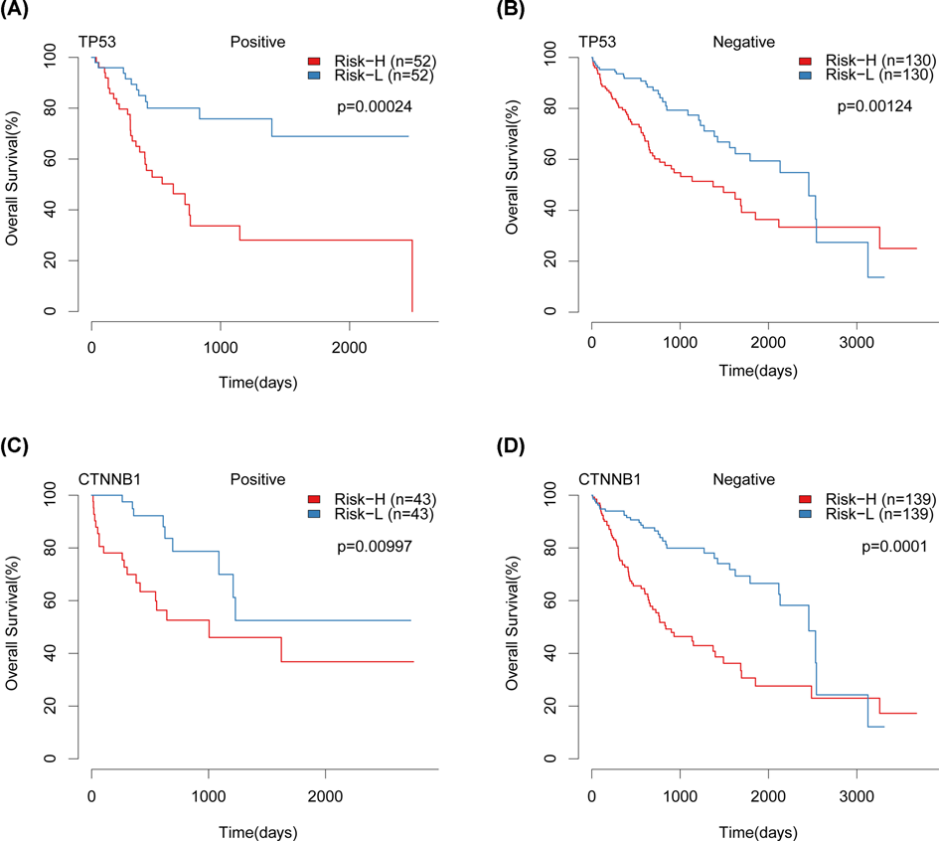
KM suvival curvers of TP53 and CTNNB1 in TCGA datasets (**A**) KM survival curves of TP53 positive samples from TCGA datasets. (**B**) KM survival curves of TP53 negative samples from TCGA data sets. (**C**) KM survival curves of CTNNB1 positive samples in TCGA data sets. (**D**) KM survival curve of CTNNB1 negative samples in TCGA datasets.

To validate the effect of our model on different subtypes of HCC, all data sets for TCGA were classified according to different SNV types, and our model was used to plot survival curves for samples of different mutation types. There was a significant difference between the high risk and the low risk group in the sample with TP53 mutation and the sample without TP53 mutation (Figure 9A,B). There were significant differences between the high risk and low risk groups in the CTNNB1 mutation and the non-CTNNB1 mutation (Figure 9C,D). These results indicated that our risk model has a good prognostic effect in different stages and subtypes.

### Comparison of risk models with other models

**Figure 10:**
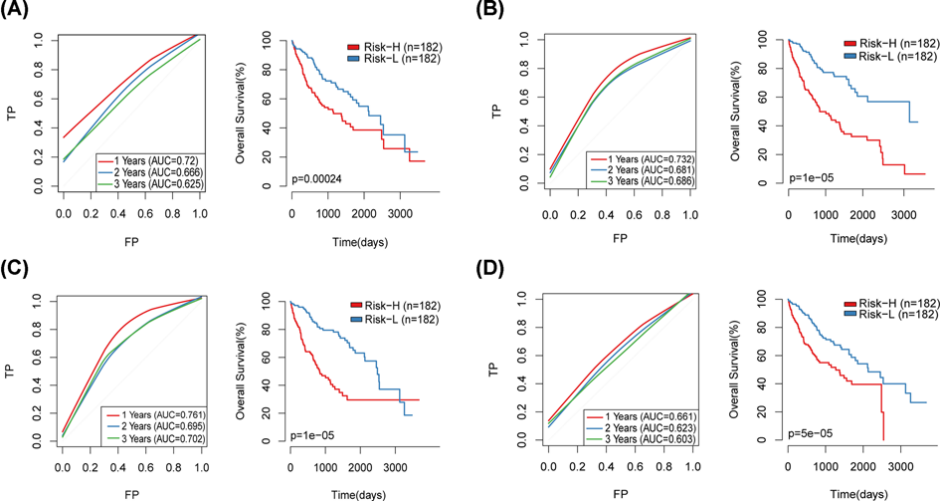
Comparison of risk models with other models (**A**) ROC and KM curves of the six-gene signature (Liu) risk model. (**B**) ROC and KM curves of eight-gene signature (Qiao). (**C**) ROC and KM curves of six-gene-based prognostic signature (Wang). (**D**) ROC and KM curves of four-gene signature (Zheng).

Four prognostic-related risk models were selected: six-gene signature (Liu) [19], eight-gene signature (Qiao) [20], six-gene-based prognostic signature (Wang) [21] and four-gene signature (Zheng) [22] were used to compare with our 10-genes model. In order to make the model comparable, the Risk score of each LIHC sample in TCGA was calculated with the same method, ROC of each model was evaluated, and the samples were divided into risk-h and risk-l groups according to the median Risk score, and the difference in OS prognosis between the two groups of samples was calculated (Figure 10). Except that the ROC curve of the four-gene signature (Zheng) model is poor (Figure 10D), the other three models have better ROC, but the average AUC of 1, 2, and 3 years is lower than that of average AUC of our 10-genes model (∼0.722, Figure 2C). The OS prognosis of the Risk-H and Risk-L group samples of these four models also showed significant differences (Figure 10).

**Figure 11:**
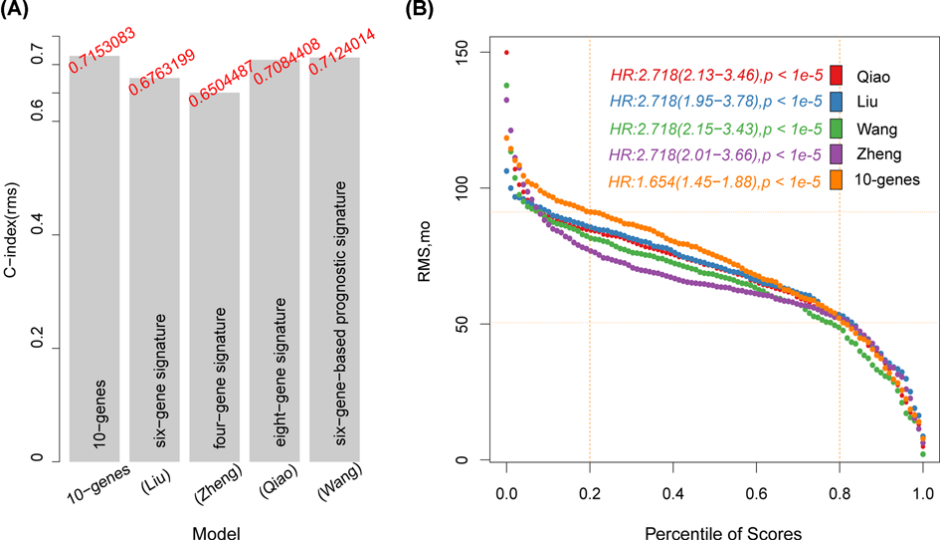
C-index and RMS of 5 prognostic risk models (**A**) C-index of five prognostic risk models. (**B**) RMS (restricted mean survival) curve of 5 prognostic risk models. The dash line represents RMS time (months) corresponding to 20% and 80% percentile score, respectively.

To compare the prediction performance of these models on LIHC samples, the RMS package in R was used to calculate 4 models and concordance index (c-index) of our model, which showed that the c-index of 10-genes model was the highest among the 5 models (Figure 11A), showing that the overall performance of the model is better than the other four. RMS time was used to evaluate the prediction effects of the five models at different time points. From the RMS curve, the five models have crossovers at 50 months. At <50 months, the Qiao, Liu, Zheng, and 10-genes risk models perform better than the Wang model (Figure 11B), This indicates that our risk model is more suitable for OS<4 years data, which can be seen from the 1, 2, and 3 years AUC values of the 5 models (Figure 2C, Figure 10).

## Discussion

We conducted a comprehensive study in which univariate and multivariate Cox regression analysis was used to identify potential immune biomarkers associated with HCC prognosis and to develop a 10-immune-related gene signature. The prognostic risk scoring system was further established and validated by test set, validation set and external data set. Immune-related genes showed good predictive value for signature of OS in HCC patients and their subgroups. It also has advantages over other genetic signatures. Together, these results provide clues for further investigation of the pathogenesis of HCC and the establishment of new risk classification and prognosis evaluation models.

Recently, research on genetic markers used to predict the prognosis of human cancers has been reported. A 4-genes signature, associated with the OS of patients with HCC, were identified and an independent prognostic factor particularly for Asian patients with serum α-fetoprotein (AFP) >20 ng/ml [23]. An immune-related gene expression pattern in liver tissues of patients with early-stage HCC that is associated with risk of HCC development in patients with cirrhosis [24]. A set of seven genes were significantly associated with overall survival (OS) for HCC and used to form a novel DNA repair-related prognostic signature [25]. An immunoscore was constructed, which contained eight immunocyte fraction types, potentially served as a candidate marker to estimate the OS for HBV-related HCC cases [26]. In our study, a 10-immune-related gene pair signature based on the TCGA dataset was developed to differentiate between high risk and low risk patients and play a good role in predicting the overall survival of HCC and its subgroups. In addition, our research suggests that RiskSocre and cell cycle, JAK-STAT significantly related to the disorder of signaling pathways that is closely related to the progress of the tumor. Our genetic model in various subtypes, stage can effectively show the prognostic value, and compared with other gene signatures. Our model has the advantages of higher are more likely to clinical application, provide service for patients.

In the present study, 10 genes related to prognosis of HCC were identified (KIF20A, CCNB1, RRAGC, DHX34, PLOD2, CDC42EP2, FEN1, DNAJC1, FTL, STAT6) and 10 immune-related gene signatures were formed. Studies have shown that Aberrant KIF20A expression might independently predict poor overall survival and recurrence-free survival of hepatocellular carcinoma [27]. CCNB1 is a cycle-like protein that regulates cell cycle G2/M, and its expression imbalance is one of the causes of malignant tumor proliferation, including HCC [28]. Researches show that disease-free survival in the high PLOD2 expression group of HCC patients was significantly shorter when compared with the low-expression group [29]. FEN1 was down-regulated in HCC tissues [30]. FTL, which is the metabolism of lipids and proteins, is increased in the presence of HCC [31]. STAT6, which is one of the members of the STAT pathway, is one of the clinical significance indicators of HCC [32]. RRAGC, DHX34, CDC42EP2 and DNAJC1 have not been reported in HCC studies. However, to our knowledge, no previous studies using these methods in HCC have focused on immune-related gene signatures with prognostic value.

Our study has some limitations. First, our findings are based entirely on a public database using bioinformatics analysis, so functional experiments are needed to verify these results. Second, the prognostic predictive value of 10 immune-related genetic markers is only based on a single cohort with a relatively small sample size, so future studies involving large independent cohorts should be conducted to verify our findings. In addition, we did not consider common clinical parameters, which may result in important information being ignored.

In conclusion, we conducted a comprehensive study to develop a signature of 10 immune-related genes, which have never been integrated before, from the TCGA database for prediction of the prognosis of HCC patients. After data processing and positive screening, a total of 536 key immune-related genes were first selected. In addition, univariate and multivariate Cox survival analysis determined the characteristics of 10 immune-related genes with prognostic function. Based on the characteristics of these 10 immune-related genes, patients in the high-risk group had significantly lower OS than those in the low-risk group.

## Supplementary Material

Supplementary Figures S1-S4

## Abbreviations

AFP: α-fetoprotein
AUC: area under the curve
GEO: Gene Expression Omnibus
HCC: hepatocellular carcinoma
IRG: immune-related gene
OS: overall suvival
TCGA: The Cancer Genome Atlas
