# Supplementary material for: Identification of hepatocellular carcinoma prognostic markers based on 10-immune gene signature
Source: Biosci Rep. 2020 Aug 28;40(8):BSR20200894. doi: 10.1042/BSR20200894 (PMC7457228; doi:10.1042/BSR20200894)
Supplement: Supplementary Figures S1-S4 [file BSR-2020-0894_supp.pdf]

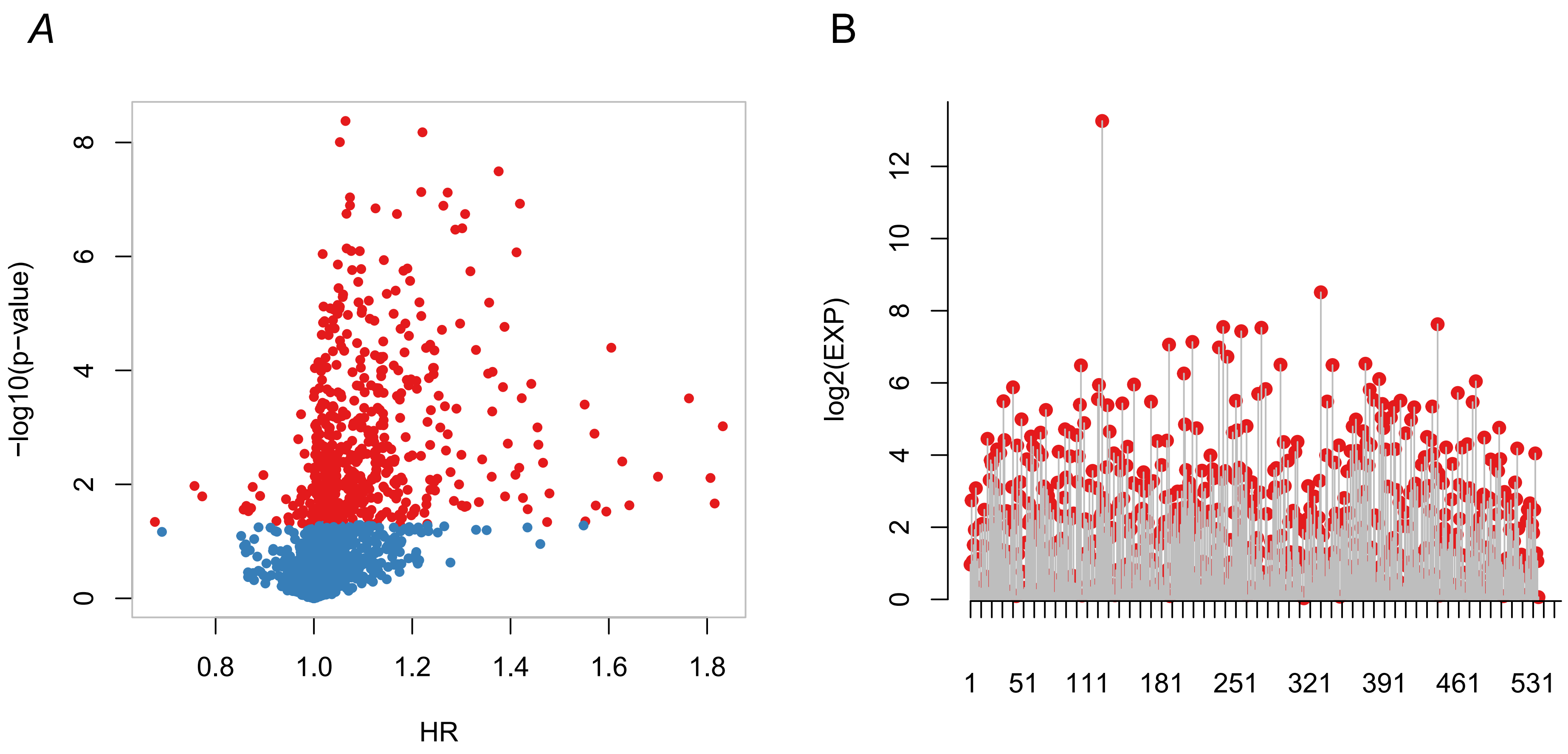

Supplementary Figure 1

Identification of differentially expressed genes. A: Differential expression using univariate regression cox analysis. The horizontal axis is the gene risk ratio HR, the vertical axis is the -log10(P) of this gene. B: Differential expression analysis of genes. The horizontal axis represents the gene, and the vertical axis represents gene expression.

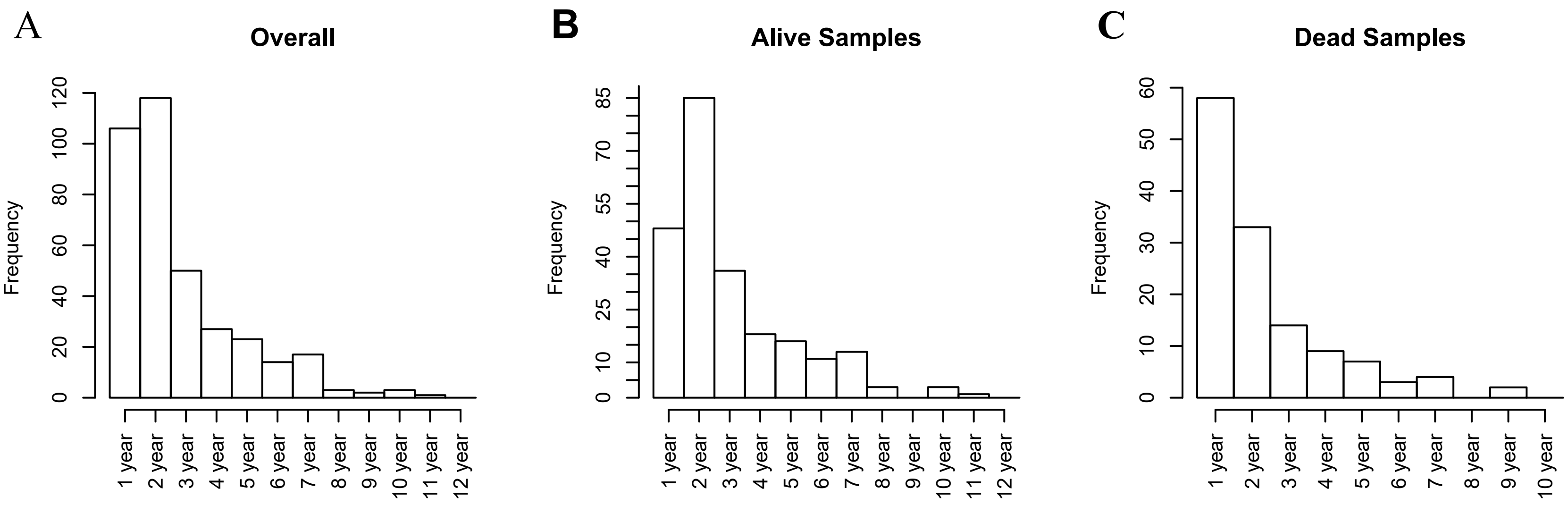

Supplementary Figure 2

Patient survival rate. A: Overall survival of all patients. B: Overall survival of alive patients. C: Overall survival of dead patients.

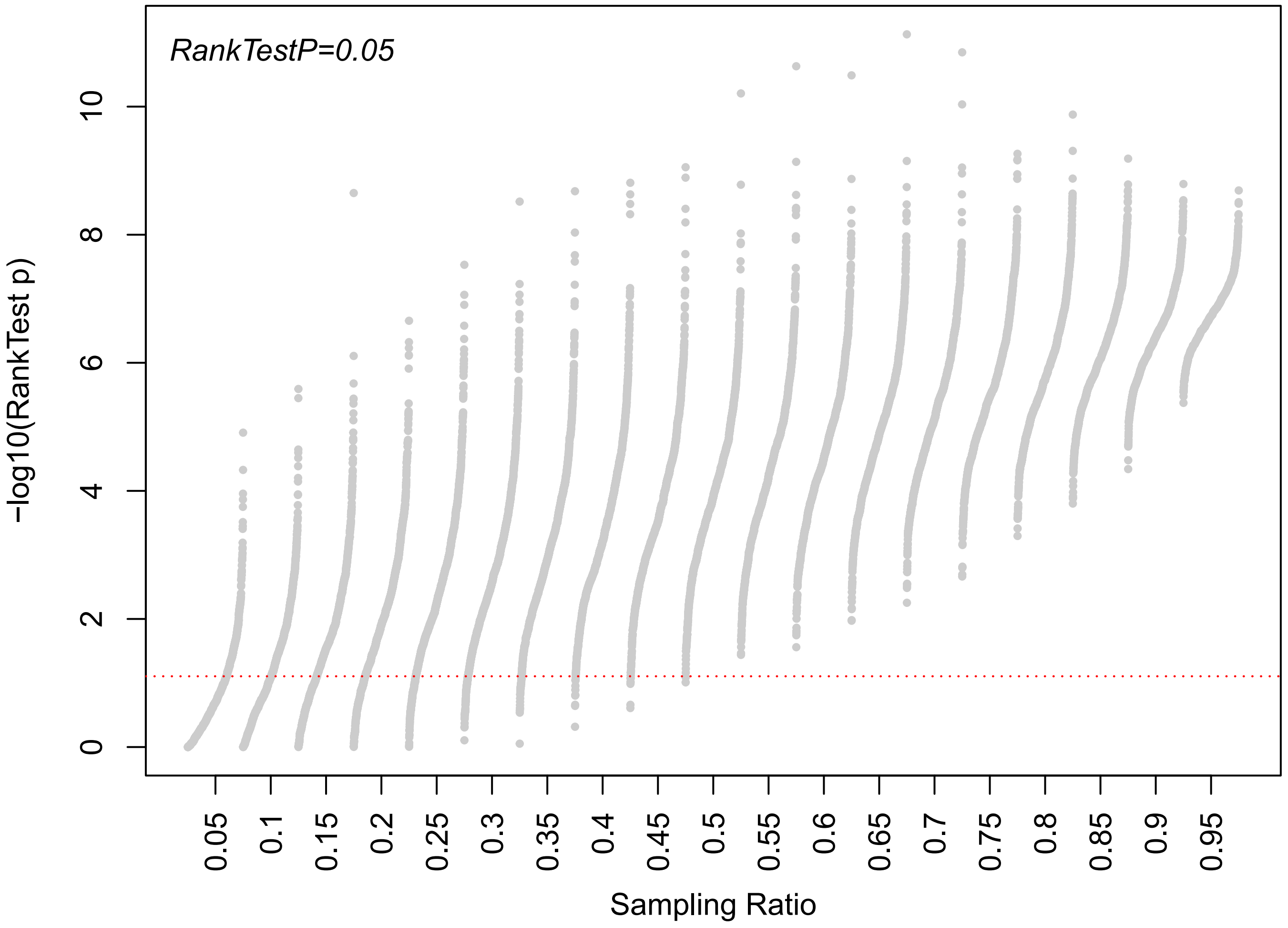

Supplementary Figure 3

Sampling ratio of 995 out of 1000 random samples.

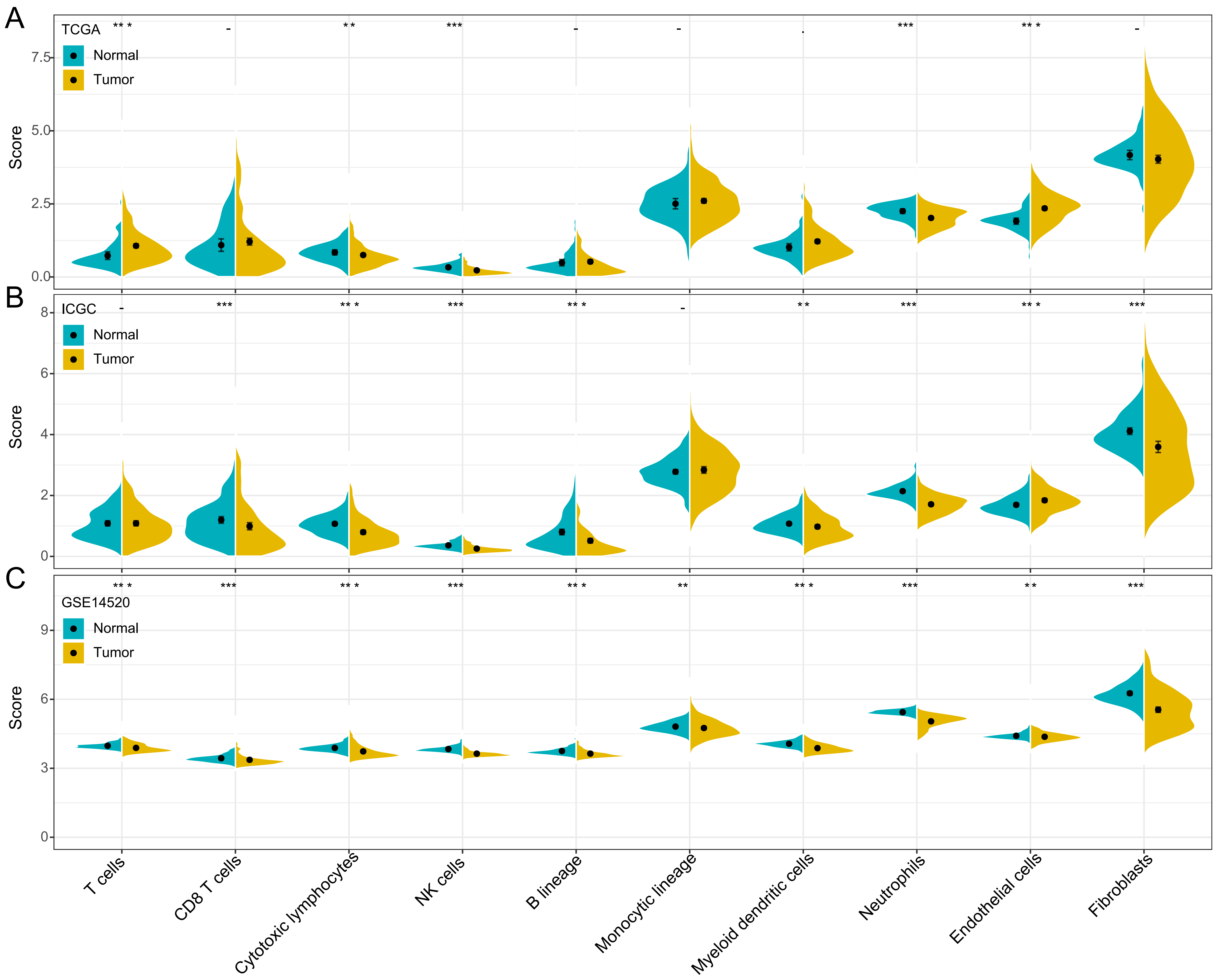

Supplementary Figure 4
The scores of 10 immune cells from the data sets TCGA, GSE14520 and ICGC-LIRI-LP.
A: Scores of 10 immune cells in in TCGA data set. B: Scores of 10 immune cells in in ICGC data set. C: Scores of 10 immune cells in GSE14520 data set.
